# Weak synchronization can alter circadian period length: implications for aging and disease conditions

**DOI:** 10.3389/fnins.2023.1242800

**Published:** 2023-09-27

**Authors:** Jihwan Myung, Sungho Hong, Christoph Schmal, Hélène Vitet, Mei-Yi Wu

**Affiliations:** ^1^Graduate Institute of Mind, Brain and Consciousness (GIMBC), Taipei Medical University, Taipei City, Taiwan; ^2^Brain and Consciousness Research Centre (BCRC), TMU-Shuang Ho Hospital, New Taipei City, Taiwan; ^3^Computational Neuroscience Unit, Okinawa Institute of Science and Technology, Okinawa, Japan; ^4^Institute for Theoretical Biology, Humboldt-Universität zu Berlin, Berlin, Germany; ^5^Department of Pediatrics, College of Medicine, National Cheng Kung University, Tainan City, Taiwan; ^6^Division of Nephrology, Department of Internal Medicine, Taipei Medical University-Shuang Ho Hospital, New Taipei City, Taiwan; ^7^Division of Nephrology, Department of Internal Medicine, School of Medicine, College of Medicine, Taipei Medical University, Taipei, Taiwan; ^8^Institute of Epidemiology and Preventive Medicine, College of Public Health, National Taiwan University, Taipei, Taiwan; ^9^TMU Research Center of Urology and Kidney, Taipei Medical University, Taipei, Taiwan

**Keywords:** unsynchronized states, period-frequency relation, circadian rhythms, frequency synchronization, Kuramoto model, period distribution, macroscopic period, mean internal period

## Abstract

The synchronization of multiple oscillators serves as the central mechanism for maintaining stable circadian rhythms in physiology and behavior. Aging and disease can disrupt synchronization, leading to changes in the periodicity of circadian activities. While our understanding of the circadian clock under synchronization has advanced significantly, less is known about its behavior outside synchronization, which can also fall within a predictable domain. These states not only impact the stability of the rhythms but also modulate the period length. In C57BL/6 mice, aging, diseases, and removal of peripheral circadian oscillators often result in lengthened behavioral circadian periods. Here, we show that these changes can be explained by a surprisingly simple mathematical relationship: the frequency is the reciprocal of the period, and its distribution becomes skewed when the period distribution is symmetric. The synchronized frequency of a population in the skewed distribution and the macroscopic frequency of combined oscillators differ, accounting for some of the atypical circadian period outputs observed in networks without synchronization. Building on this finding, we investigate the dynamics of circadian outputs in the context of aging and disease, where synchronization is weakened.

## Introduction

Animals innately follow a near-24-h cycle of rest and activity, known as the circadian rhythm, which prepares them for daily environmental changes. The endogenous rhythm in behavioral activities is maintained with remarkable precision under constant darkness, exhibiting robust periodicity over months and minimal cycle-to-cycle variation in activity phase (Pittendrigh and Daan, 1976a; Schwartz and Zimmerman, 1990). In mammals, the suprachiasmatic nucleus (SCN) serves as the central clock, orchestrating both behavioral circadian rhythms and physiological rhythms throughout the body. The SCN is a network of circadian oscillators, with single neurons and glial cells as the cellular identities, that maintain rhythmic expressions of circadian clock molecules through the transcription-translation feedback loop (TTFL).

The oscillation within a single cell is both autonomous and persistent, yet displays a variation in period across the population (Leise et al., 2012). These oscillators couple within the network to generate a synchronized oscillation, reducing period heterogeneity and facilitating high temporal precision for the circadian clock output at the organismal level (Herzog et al., 2004). The synchronization is the essential mechanism of the SCN network that transforms diverse period, phase, and amplitude of individual oscillators into predictable and coherent outputs. However, biological systems often operate in the metastable state between complete synchronization and desynchronization (Kelso, 1995). This is sometimes due to the functional needs, such as the internal representation of seasonal time within the SCN (Pittendrigh and Daan, 1976b; Myung et al., 2015; Schmal, 2023), but it can also be due to degradation of the network through aging and disruptive timing cues such as constant light (Ohta et al., 2005; Farajnia et al., 2012).

Aging is known to cause changes in the period of circadian locomotor activity (Pittendrigh and Daan, 1974). In the widely studied laboratory mouse strain C57BL, circadian activities persist through aging, with periods typically lengthening with increasing age (Davis and Menaker, 1981; Welsh et al., 1986; Possidente et al., 1995; Valentinuzzi et al., 1997). Depending on the strain and entrainment history, periods can also shorten (Pittendrigh and Daan, 1976a). Chronic illnesses often lead to changes in circadian periodicity, which can result in sleep disturbances, as seen in diseases like Alzheimer's or Huntington's (Witting et al., 1990; Aziz et al., 2010). Disruptions in circadian gene expression have been observed in animal models of chronic kidney disease (CKD) (Hsu et al., 2012). This disruption causes instability in circadian activity when in constant darkness (Myung et al., 2019). Furthermore, disturbances in circadian rhythm have been identified in conditions such as acute respiratory failure (ARF) (Yang et al., 2020) and chronic pulmonary disease (COPD) (Giri et al., 2022). In critically ill patients, there have been reports of misalignment in internal circadian rhythms (Felten et al., 2023).

Yet, within the widely recognized Kuramoto model for synchronization, explaining these changes in period remains a challenge (Acebrón et al., 2005). Emerging evidence suggests a correlation between the period and amplitude (Myung et al., 2018; del Olmo et al., 2023). As synchronization increases, so does the circadian amplitude of a clock ensemble (Schmal et al., 2018). Oscillators achieving synchronization is fundamentally about aligning their frequencies. In systems with a finite number of oscillators, it has been numerically shown that skewness in the frequency distribution can eventually alter the mean frequency of macroscopic ensemble oscillations (Peter and Pikovsky, 2018). Given the reciprocal relationship between period and frequency, we note that a symmetric period distribution, such as Gaussian, results in a skewed frequency distribution. This skewness can influence the mean period of an oscillator ensemble, particularly when the standard deviation of the period distribution is large. This may provide additional insight into the changes of circadian period under weak synchronization observed in aging and disease conditions.

## Results

### Skewed frequency distribution: mean, median, and the mode

A circadian oscillator within a single cell emerges from nonlinear molecular feedback networks that contain ultrasensitive response motifs (Zhang et al., 2013). The oscillatory trajectory is believed to follow a stable limit cycle, allowing the oscillation in the phase space to be mapped onto a unit circle. This property enables the reduction of the nonlinear oscillator to a phase oscillator which, in turn, facilitates the investigation of collective behavior of multiple oscillators (Winfree, 1980). The temporal evolution of a circadian oscillator at phase θ with constant frequency *f* (where *f* is the reciprocal of the intrinsic period, τ) can be described by a differential equation dθ/d*t* = 2π*f* = 2π/τ. The Kuramoto model extends this framework by introducing a coupling term with a sine of the phase difference (Acebrón et al., 2005). At least for a certain class of oscillators with a particular type of phase response curve (PRC), this provides a concise formalism for describing the synchronization behavior among multiple oscillators under various coupling scenarios (Myung and Pauls, 2018). Under the assumption of a symmetric distribution of the individual oscillator frequencies, the model predicts that the frequency of the synchronized ensemble is determined by the average frequency of the population, which appears true for the SCN (Liu et al., 1997).

The periods of circadian firing rates in dissociated single SCN neurons show Gaussian distribution in both rats and C57BL/6J mice (Honma et al., 2004, 2012). For the mean period τ_0_ and standard deviation σ, the Gaussian probability density function *p* is


(1) $$\begin{array}{l} p\left(\tau \right)=\frac{1}{\sqrt{2\pi \sigma^{2}}}e^{-{\left(\tau -\tau_{0}\right)}^{2}/2\sigma^{2}} \end{array}$$

which satisfies ∫*dτp*(τ)=1 .

By change of variables, the probability density function for the frequency *f* can be written as


(2) $$\begin{array}{l} q\left(f\right)=\frac{1}{\sqrt{2\pi \sigma^{2}}f^{2}}e^{-{\left(1/f-\tau_{0}\right)}^{2}/2\sigma^{2}} \end{array}$$

which has a singularity at *f* = 0.

**Figure 1:**
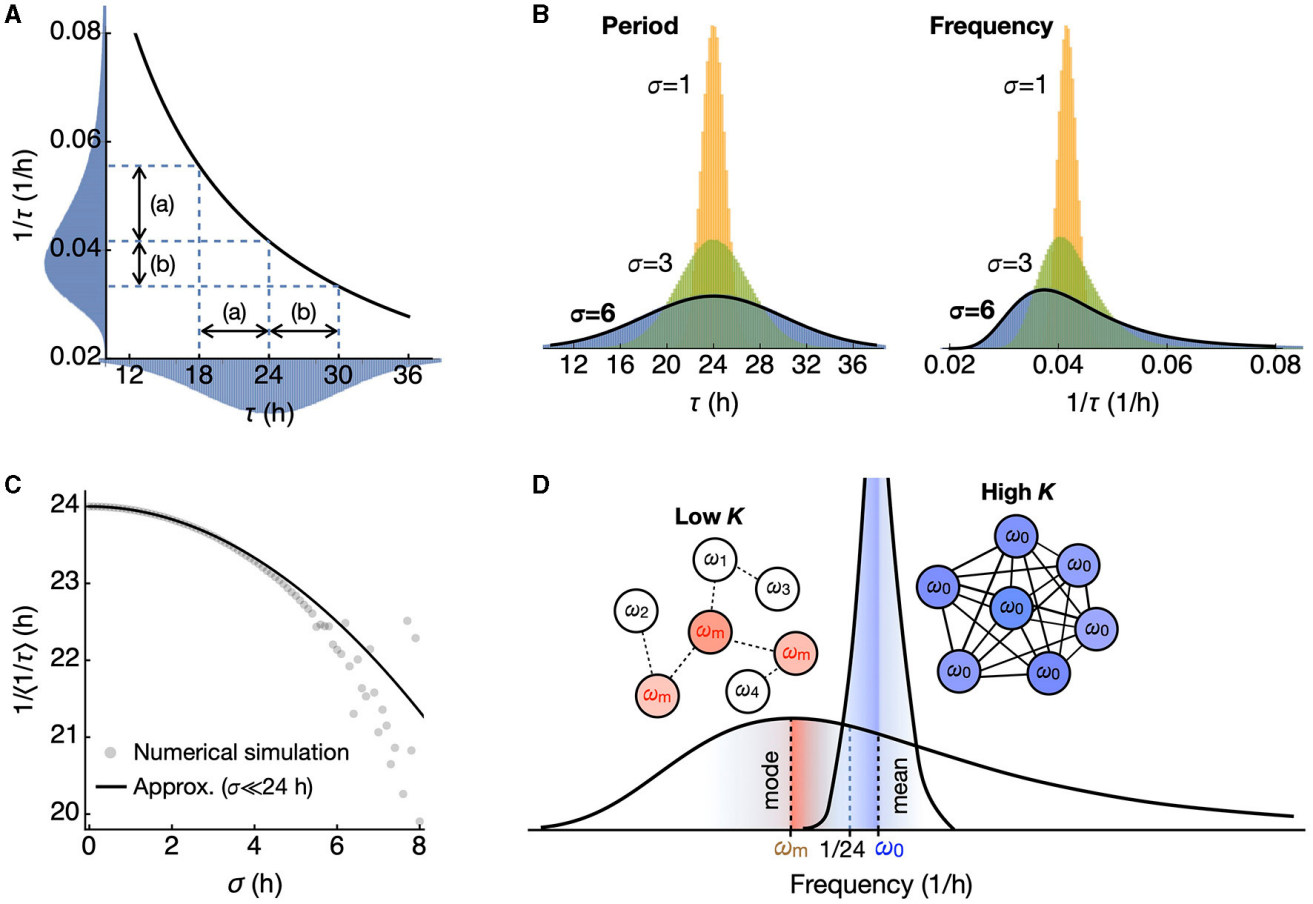
A symmetric period distribution corresponds to a right-skewed frequency distribution. **(A)** The reciprocal function connects period (τ) and frequency (1/τ). In a symmetric period distribution with a 24-h mean, shorter periods correspond to a broader frequency range (a), while longer periods correspond to a narrower range (b). This results in a right-skewed frequency distribution. **(B)** The skewness of the frequency distribution increases as the standard deviation σ of the symmetric period distribution increases. **(C)** The inverse of the mean frequency <1/τ> shows a quadratic decrease as the standard deviation σ increases. **(D)** In the skewed frequency distribution, the mode (ω_m_; red) leans toward lower frequencies (longer periods), while the mean (ω_0_; blue) leans toward higher frequencies (shorter periods), compared to 1/24 h, the inverse of the mean period. Upon oscillator coupling, synchronization occurs at the mean frequency, distinct from the inverse of the mean period.

The reciprocal transformation maps shorter periods to a wider range on the higher frequency side, resulting in a skewed distribution with a peak (mode) shifted to the lower frequency side (Figures 1A, B). The 1/*f*^2^ term in the equation (2) implies this shift, which gives higher weight to the lower frequency side. Due to the singularity, there is no simple closed-form solution for the mean <*f*> but via the Dawson function *F*,


(3) $$\begin{array}{l} \langle f\rangle =\frac{\sqrt{2}}{\sigma }F\left(\frac{\tau_{0}}{\sqrt{2}\sigma }\right)\approx \frac{1}{\tau_{0}}+\frac{\sigma^{2}}{\tau_{0}^{3}}+\frac{3\sigma^{4}}{\tau_{0}^{5}}+\;\cdots . \end{array}$$

Since we are interested in the regime σ ≪ τ_0_, the reciprocal of the mean frequency approximates to


(4) $$\begin{array}{l} 1/\langle f\rangle \approx \tau_{0}-\frac{\sigma^{2}}{\tau_{0}}. \end{array}$$

This provides a good approximation compared to the mean values of the randomly generated populations (Figure 1C) when standard deviations are small. It ensures that, for nonzero σ, the reciprocal of the mean frequency is shorter than the mean period τ_0_. This effect becomes more pronounced as σ increases. The reciprocal of the median frequency is approximately τ_0_ and can be found at the half-maximal point of the cumulative probability distribution. The reciprocal of the mode frequency, found where *q*'(τ)=0, is longer than τ_0_. In the regime where σ ≪ τ_0_ it approximates to


(5) $$\begin{array}{l} 1/f_{\text{mode}}\approx \tau_{0}+\frac{2\sigma^{2}}{\tau_{0}}. \end{array}$$

Therefore, the reciprocal of the mean frequency is the shortest, followed by the reciprocal of the median frequency, and then by the reciprocal of the mode frequency when the frequency distribution is skewed (Figure 1D). As we show later, the macroscopic period resulting from the summation of uncoupled oscillators is longer than the average of the intrinsic periods. In contrast, the reciprocal of the synchronization frequency of coupled oscillators corresponds to that of the mean frequency, as predicted by the Kuramoto model (Saha and Amritkar, 2014; Peter and Pikovsky, 2018).

### Macroscopic period of uncoupled oscillators

We begin with an extreme case of a collection of oscillators with a given frequency distribution that are uncoupled but start oscillation at the synchronized state. Then, their macroscopic oscillation is the result of integrating each oscillator multiplied by its probability density as


(6) $$\begin{array}{l} I=\int d\tau e^{-{\left(\tau -\tau_{0}\right)}^{2}/2\sigma^{2}}\cdot e^{2\pi it/\tau }. \end{array}$$

However, this integration is not straightforward for the period distribution because the period τ appears in the denominator, leading to a singularity. We can find an approximate expression for small σ ≪ τ_0_ using the steepest descent method (see Materials and Methods).


(7) $$\begin{array}{l} I\approx \frac{1}{\sqrt{2\pi \sigma^{2}}}\exp\left[\frac{2\pi i}{\tau_{0}}t-\frac{2\pi^{2}\sigma^{2}}{\tau_{0}^{4}}t^{2}-\frac{8\pi^{3}\sigma^{4}i}{\tau_{0}^{7}}t^{3}\right] \end{array}$$

**Figure 2:**
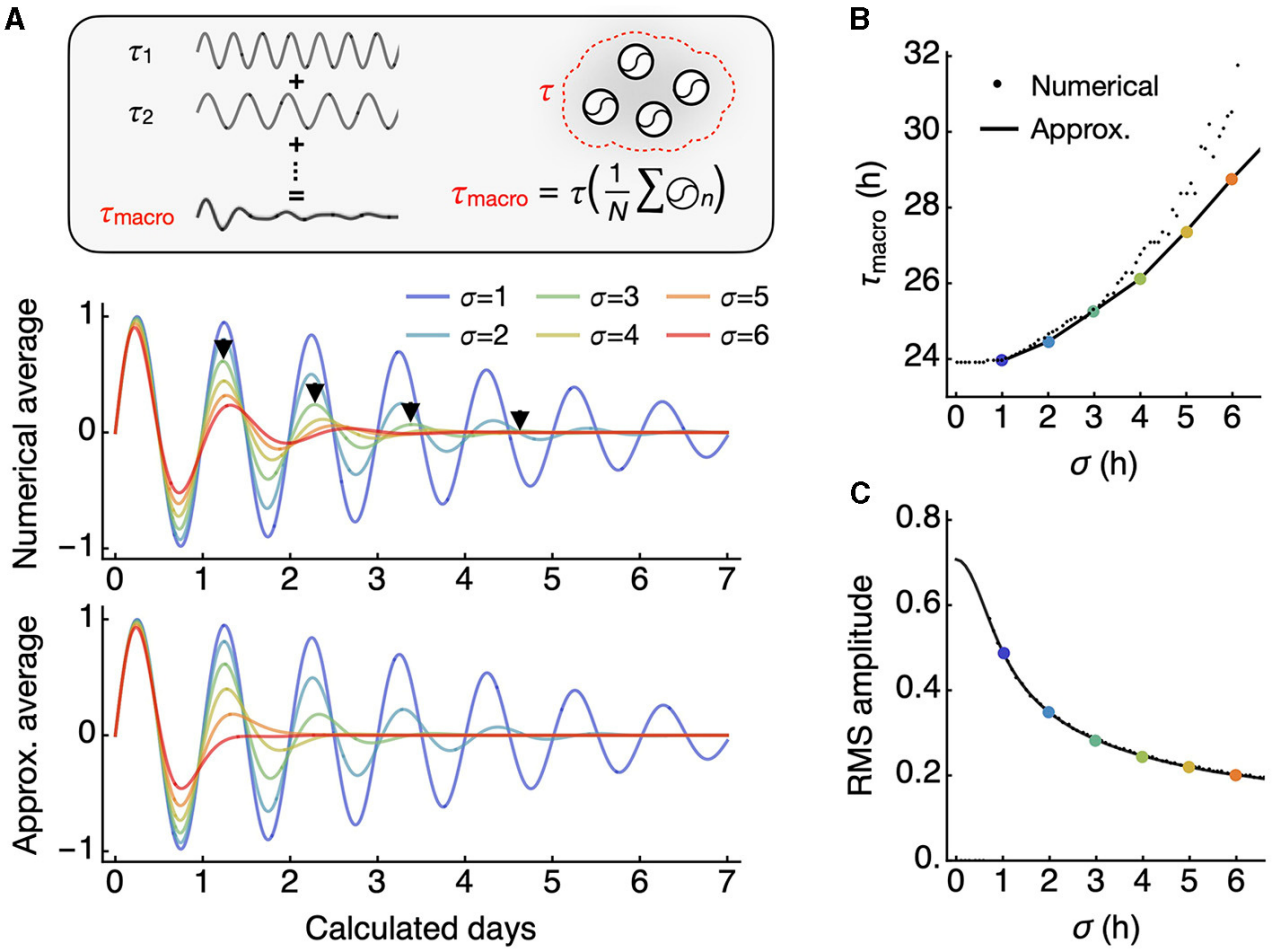
The macroscopic oscillation period is longer than the mean of individual oscillator periods. **(A)** The macroscopic oscillation represents the whole-field average of all individual oscillators (inset). The numerical integration of all oscillators over a continuous distribution results in a damped macroscopic oscillation with an increasing period, as indicated by each arrowhead marking peak positions (middle). The integral has an approximation in closed form that also shows an increasing period (bottom). **(B)** As the standard deviation of the period distribution increases, there is a corresponding increase in the macroscopic period. For smaller standard deviations, the analytic approximation (line) aligns closely with the numerical averages (small dots). **(C)** The root mean square (RMS) amplitude decreases with an increasing standard deviation, in an inverse relationship. The approximation (line) closely predicts the numerical averages (small dots).

Therefore, with higher σ, the macroscopic oscillation *I* damps while its period increases. If their frequencies, not periods, were from a Gaussian distribution, we would still see dampening, but the period would remain the same. This result is confirmed by numerical integration compared to the approximation (Eq. 7) (Figure 2A). Both the period of *I* (the macroscopic period) and the root mean square (RMS; square root of mean of squared values) as collective amplitude of *I* align well with the approximation (Figures 2B, C).

### Synchronization period through increased coupling

In the other case, increasing coupling drives oscillators toward synchronized oscillation with the mean frequency. On its course, the discrepancy between the macroscopic period and the mean period in the distribution of individual oscillators narrows. With the coupling strength *K*, the evolution of phase θ_*i*_ in each oscillator is described by the following equation:


(8) $$\begin{array}{l} \frac{d\theta_{i}}{dt}=\frac{2\pi }{\tau_{i}}+\frac{K}{N}\overset{N}{\underset{j}{\sum }}\sin\left(\theta_{j}-\theta_{i}\right) \end{array}$$

**Figure 3:**
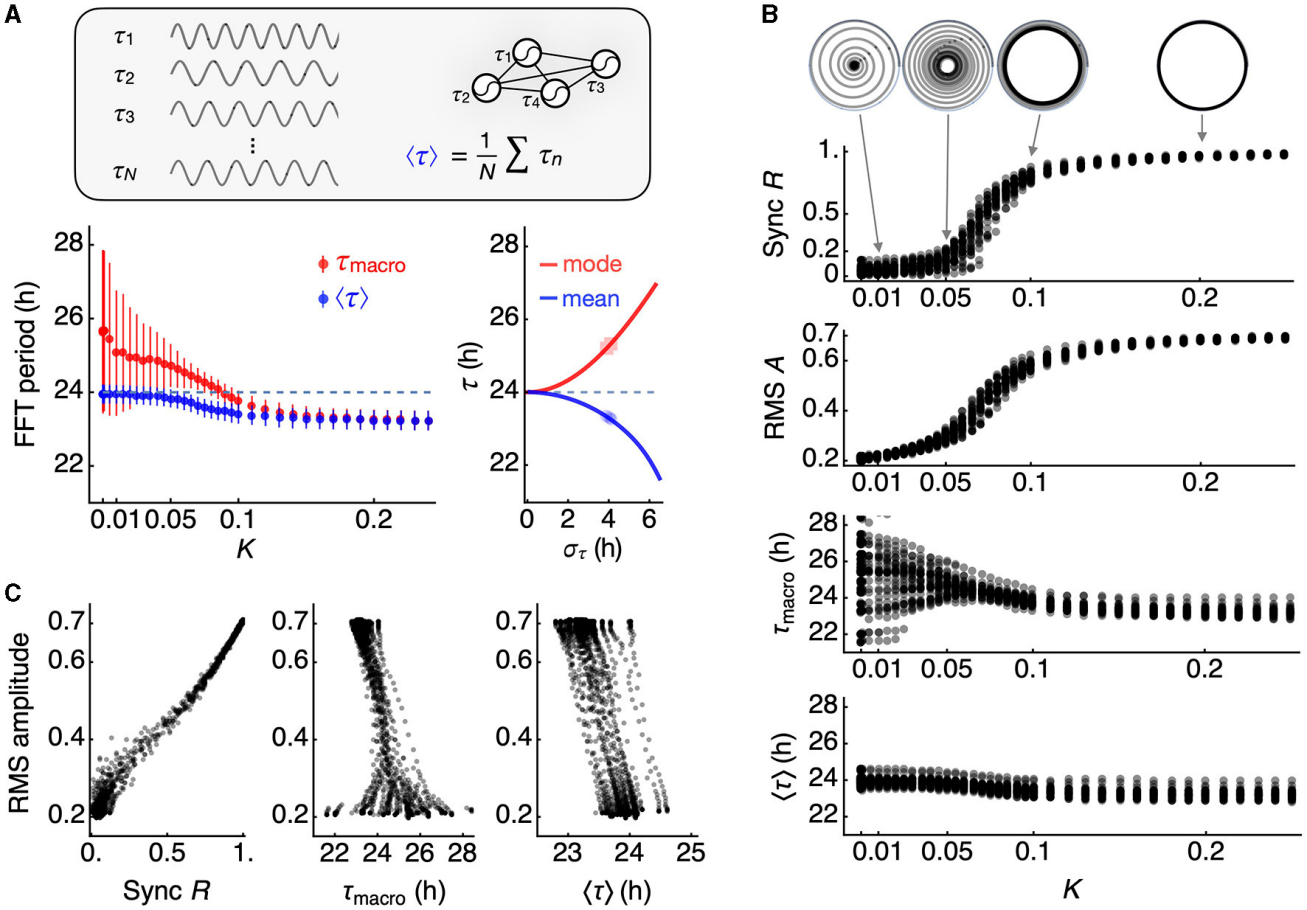
Changes in macroscopic circadian parameters under network coupling. **(A)** The mean of individual oscillator periods (inset), estimated by FFT (blue datapoints), differs from the macroscopic oscillation period (red datapoints). At a low coupling constant *K*, the mean of individual oscillator periods (24 h, dashed line) follows the inverse of the mean frequency, in contrast with the macroscopic period of the mean oscillation. As network coupling strengthens, these two estimates of periods converge (left panel). The error bars represent the standard deviation across 30 simulated networks. When coupling falls below a critical level for synchronization, the mode of the frequency distribution predicts the macroscopic period, while the mean of the frequency distribution predicts the mean of individual periods (right panel). The periods of 300 individual oscillators are randomly selected from 30 normal distributions with a standard deviation of 4 h. **(B)** Synchronization emerges in a switch-like fashion with increasing coupling strength. The collective phase evolves over time under each coupling condition but settles into a collective amplitude *R*, known as the Kuramoto order parameter (top), and the RMS amplitude of the macroscopic oscillation closely follows *R* (second from top). The macroscopic period is variable when the coupling is below a critical level (third from top), while the mean of individual oscillator periods is less variable (bottom). Both estimates of periods converge to a period shorter than the mean period in the original period distribution. **(C)** The order parameter *R* shows a strong correlation with the RMS amplitude (correlation coefficient = 0.996 ± 0.002 for 30 network simulations) (left panel). A generally negative relationship is observed between the macroscopic period and the RMS amplitude (correlation coefficient = −0.770 ± 0.313 for 30 network simulations) (mid panel), while the mean of oscillator periods exhibits a perfect negative correlation (-0.997 ± 0.002 for 30 network simulations) (right panel).

We generated 30 simulated networks, each consisting of 300 oscillators with a Gaussian period distribution (σ = 4 h). When the coupling strength (*K*) is below the critical level, the macroscopic period tends to be longer than the mean period on average, and this can be accurately predicted by the reciprocal of the mode frequency (Figure 3A, red). However, as *K* exceeds the critical level (approximately 0.1 in this case), synchronization occurs at a period shorter than the median (24 h). The reciprocal of the mean frequency provides a good estimate of the synchronized frequency for the given period distribution (Figure 3A, blue).

We present the simulation results for all 30 networks in Figure 3B. The Kuramoto order parameter *R* indicates the degree of synchronization of the oscillators at each level of *K*. Notably, *R* evolves into a stable orbit, even when *R* is below 1 (Figure 3B, inset). We note that the RMS amplitude serves as a good indicator of *R*, reflecting the switch-like characteristic of *R* with respect to *K*. In the absence of coupling, the macroscopic period of *I* can vary, but both the macroscopic period and the mean period converge toward a shorter period, as we have described. This results in an inverse correlation between the average period and the RMS amplitude (Figure 3C), a relationship reminiscent of the twist relationship observed in the choroid plexus (Myung et al., 2018). This relationship effectively captures the broader impacts of synchronization, given that *R* is proportional to the RMS amplitude.

## Discussion

It is plausible that, within the mammalian circadian system, coupling and the resulting synchronization are integral components of its design. Most discussions on synchronization assume a Gaussian distribution of periods, where synchronization occurs at the mean period. However, the formalism of the Kuramoto model predicts that synchronization is achieved at the mean frequency. This distinction might seem subtle but could carry significant implications.

The effects of the skewed frequency distribution due to period variance that we explored are for σ = 4 h. Data on the electrical firing rate and the bioluminescent clock gene reporter activity from dissociated single SCN neurons show a standard deviation of <2 h (Herzog et al., 2004; Honma et al., 2004, 2012). This amounts to <10% difference between the mean and median frequencies. This difference diminishes as the synchronization tightens the distribution (Figure 1D). When the system is coupled below the critical level, the effects of a skewed frequency distribution become apparent in externally observable states of period and amplitude. Specifically, the period systematically deviates from the mean period. Our analytical and numerical approach provides insights into these deviations.

The effect described above is closely related to another issue in chronobiology, namely the skewed Arnold tongue when presented in terms of period instead of frequency (Schmal et al., 2015, 2020). The simplest form of synchronization, commonly referred to as entrainment, is the unidirectional synchronization of an internal clock to an external Zeitgeber signal, such as rhythmic light or temperature cues. Entrainment typically occurs within a wedge-shaped entrainment region within the Zeitgeber period (*T*) and Zeitgeber strength (*K*) parameter plane that broadens for large Zeitgeber strength and tapers toward the intrinsic period (τ) for decreasing strength *K*. Since the information of the Zeitgeber signal typically enters the underlying system's equations via the frequency, the reciprocal relationship between frequency and period leads to an asymmetric entrainment region in the period domain. This has implications for circadian physiology as it could directly translate into asymmetric distributions of chronotypes.

**Figure 4:**
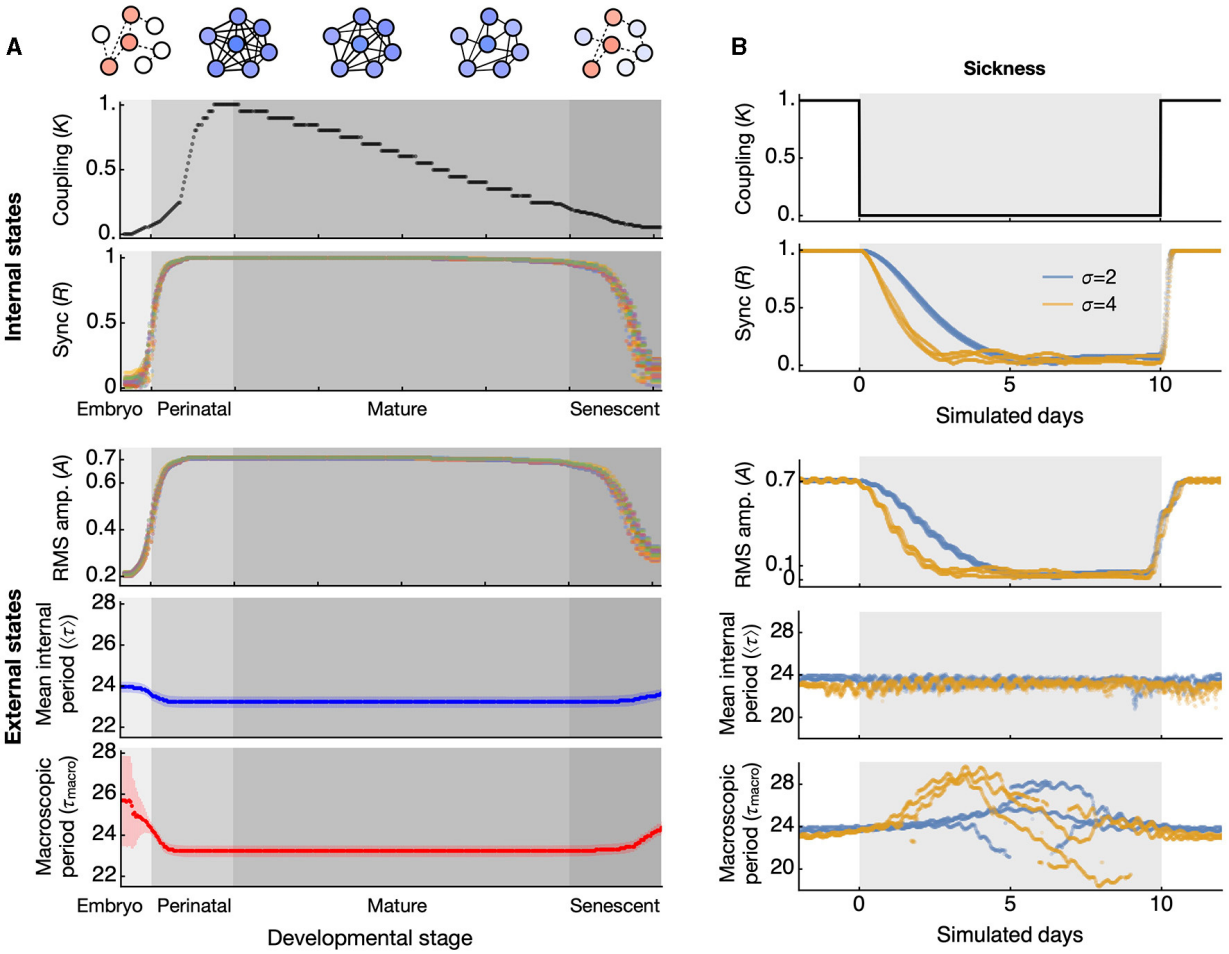
Simulated test of synchronization-induced amplitude and period alterations at the whole-animal level in aging and disease conditions. **(A)** Throughout development and aging, the coupling of the circadian clock network is hypothesized to increase and mature at the end of the perinatal stage, then gradually decrease until senescence (top panel). The synchronization of circadian clocks, quantified by the order parameter *R*, shows a discrete transition of states (second from top). These two are internal states that cannot be directly observed. The externally observable circadian rhythms alter according to the state of synchronization. The amplitude of circadian activity correlates positively with the synchronization state (third from top). The datapoints in different colors represent simulated data from 30 different networks, each composed of 300 oscillators randomly selected from period distributions with a standard deviation of 4 h. The period generally correlates negatively with the synchronization state, with less variation in the mean period (blue, fourth from top) than in the macroscopic period (red, bottom panel). The error bars indicate the standard deviation across 30 networks. **(B)** Under abrupt changes in coupling strength, which can occur during acute disease conditions (top panel), the degree of synchronization gradually decreases during the transition (second from top). This transitional change is reflected in the RMS amplitude of circadian activity (third from top). The mean of all oscillator periods slightly increases during the abrupt drop in coupling (fourth from top), while the macroscopic period changes drastically during the same drop in coupling (bottom). The periods indicated are evaluated from a 5-day sliding window FFT. The datapoints in pale blue and light orange colors represent results from simulations of three networks each, derived from two distributions with standard deviations of 2 and 4 h, respectively.

In a broader biological context, our findings have potential implications for both development and disease. It is believed that the coupling within the circadian clock network changes throughout development (Olejniczak et al., 2023). Around the time of birth, the circadian period displays wide variations across species (Rivkees, 2003; Yamazaki et al., 2005; Bellavia et al., 2006), a phenomenon potentially explained by the lack of coupling (Weinert and Weiß, 1997). As aging progresses, the clock network is thought to deteriorate (Farajnia et al., 2014). Thus, the network coupling, established during embryonic and perinatal stages, might peak and then gradually decline throughout mature and senescent stages (Figure 4A, upper, “Internal states”). Although this deterioration is gradual, the switch-like relationship between coupling strength (*K*) and synchronization (*R*) implies that *R* will remain stable as long as *K* stays above a critical level. As senescence begins and coupling weakens, there may be stage-specific alterations in circadian amplitude and periods (Figure 4A, lower, “External states”). In humans, these senescence-related changes are evident among Alzheimer's disease (AD) patients, with low circadian activity amplitude and delayed acrophase (Satlin et al., 1995).

Similarly, a sudden illness will not have immediate effects on the circadian rhythm since the underlying oscillators have already been well synchronized, and they require time to gradually fall into desynchronization (Myung et al., 2012). Even if an illness were to completely disrupt the coupling, the RMS amplitude would decay gradually and the mean period would remain initially unaffected (Figure 4B). However, over time the macroscopic period will experience significant fluctuations, a phenomenon consistent with the unstable circadian period observed in chronic illnesses, such as CKD (Myung et al., 2019). A significant alteration in circadian period is also observed in AD (Volicer et al., 2012).

There is a question concerning the interpretation of the macroscopic period (τ_macro_) compared to the mean internal period (τ_0_). This question ties into a longstanding debate within SCN physiology regarding the preferred extracellular signal pathway: diffusive signaling (also known as volume transmission) vs. synaptic signaling (also known as wiring transmission) (Moore, 2013). The macroscopic period is estimated from the average oscillation of the entire ensemble. In the bioluminescent reporter system, this value corresponds to the whole-field luminometry data from a culture dish. The signal through volume transmission would carry information of the macroscopic period as it is the average of the entire ensemble output. In contrast, the mean internal period is calculated as the average period of each individual cellular oscillator, as determined from imaging data. From a distant tissue receiving the circadian signal through volume transmission, the detailed individual activities are unknown. Synaptic signaling originates from individual neurons, and therefore, individual periods can be accessed. In this context, the macroscopic period corresponds to the period observed in volume transmission, while the mean internal period can be evaluated through synaptic signaling. The circadian amplitude of volume transmission reflects the RMS amplitude of the clock assembly *I*, whereas the amplitude of wiring transmission reflects the order parameter *R*. These two are comparable, as seen in Figure 3. However, the variation of period at low coupling strength is much larger in the volume transmission (as reflected by the macroscopic period) than in the synaptic transmission (reflected by the mean internal period). At least for the circadian locomotor outputs, the macroscopic period from our simulation seems to better represent realistic observations, where the diffusible clock signals originate in the SCN (LeSauter and Silver, 1998) and propagate through cerebrospinal fluid irrigation (Leak and Moore, 2012).

In this study, we demonstrated that synchronization can influence the observed period of an ensemble. One potential application is assessing the synchronization state of the cell population by examining the macroscopic period of total reporter activities from cultured cells or tissues. The degree of synchronization can vary due to factors such as development, the level of integration within a tissue, or pathological conditions. Therefore, synchronization can serve as a qualitative indicator of these states, which can be gauged by the macroscopic period and/or period distribution. In principle, it is possible to evaluate the phenotype of an *in vitro* culture reflecting these states (Kumpošt et al., 2021). Although obtaining an exact measure of synchronization might be challenging, as indicated in Figure 4A, the macroscopic period can provide insights into developmental maturation, cellular interactions, or even the pathological phenotype of cellular ensembles modeling a disease. This becomes particularly relevant when individual periods are not directly accessible. Since synchronization can influence the macroscopic period, such determinations can be made solely by observing this period. This approach can be especially valuable in studies, for example, of spheroids.

Although the coupling strength may not be directly measurable in a given individual, our study suggests that other observable features could indicate decreased coupling. For instance, a decrease in amplitude of activities and core temperature can signal a loss of synchrony and/or coupling. It is also conceivable that the changes in the circadian period caused by decreased coupling could lead to a desynchronization of behavioral organization within the same individual, such as timings of eating and motor behaviors. These potential indicators could be used to evaluate the loss of internal synchrony or coupling, which could in turn inform the development of personalized chronotherapies, an approach that has yet to demonstrate significant benefits (Lee et al., 2021).

It is interesting to note that in our schematic simulation over the course of life, the critical coupling level to enter or exit the stable period would be crossed twice. However, the components of the network can change through aging (Farajnia et al., 2014), and it is unclear whether the critical coupling strength at these two points corresponds to the same value. It would also be important to note that the environmental factors can affect differently toward synchronization at these two points of development. These factors can be systemic, given that other peripheral clocks can influence the pacing of the master clock (Myung et al., 2018, 2019; Chrobok et al., 2022). Therefore, it would be valuable to investigate what other factors, in conjunction with the local network coupling, determine the fate of synchrony.

## Materials and methods

### Approximate mean period of the skewed frequency distribution

If the period follows a Gaussian distribution with mean τ_0_, and variance σ^2^, a generating function can be defined as follows.


$$\begin{array}{l} Z\left[\omega \right]=\int \frac{d\tau }{\sqrt{2\pi \sigma^{2}}}e^{-{\left(\tau -\tau_{0}\right)}^{2}/2\sigma^{2}+\omega \tau }. \end{array}$$

Then Z[ω] satisfies


$$\begin{array}{l} \int d\tau p\left(\tau \right)=Z\left[\omega =0\right]=1\\ \langle \tau \rangle =\partial_{\omega }Z\left[\omega =0\right]=\tau_{0}\\ \langle \tau^{2}\rangle =\partial_{\omega }^{2}Z\left[\omega =0\right]=\tau_{0}^{2}+\sigma^{2}. \end{array}$$

Here we define another function


$$\begin{array}{l} Y\left[\omega \right]=\int \frac{d\tau }{\sqrt{2\pi \sigma^{2}}}\cdot \frac{1}{\tau }\cdot e^{-{\left(\tau -\tau_{0}\right)}^{2}/2\sigma^{2}+\omega \tau }, \end{array}$$

such that ∂_ω_*Y*[ω]=*Z*[ω] .

By direct integration, we get


$$\begin{array}{l} Z\left[\omega \right]=\exp\left[-\frac{\tau_{0}}{2\sigma^{2}}+\frac{\sigma^{2}}{2}{\left(\omega +\tau_{0}/\sigma^{2}\right)}^{2}\right]. \end{array}$$

Using this,


$$\begin{array}{l} \;Y\left[\omega \right]=\int d\omega Z\left[\omega \right]=\frac{\sqrt{2}}{\sigma }e^{\tau_{0}\omega +\frac{\sigma^{2}\omega^{2}}{2}}F\left(\frac{\tau_{0}+\sigma^{2}\omega }{\sqrt{2}\sigma }\right)+C\;, \end{array}$$

where *F* is the Dawson function. *C* = 0 will become evident later. Then,


$$\begin{array}{l} \;\langle \frac{1}{\tau }\rangle =Y\left[0\right]=\frac{\sqrt{2}}{\sigma }F\left(\frac{\tau_{0}}{\sqrt{2}\sigma }\right). \end{array}$$

which gives equation (3).

### Approximate expression for the macroscopic oscillator *I*

We use the steepest descent method for approximation (Strogatz, 2014). Equation (6) can be re-written for simplification such that


$$\begin{array}{l} \;I=\int d\tau e^{-L\left(\tau \right)/2\sigma^{2}} \end{array}$$

where


$$\begin{array}{l} L\left(\tau \right)={\left(\tau -\tau_{0}\right)}^{2}-2i\alpha \sigma^{2}\tau^{-1},\;\alpha =2\pi t \end{array}$$

We find the stationary point by *L*'(τ) = 0 and therefore,


$$\begin{array}{l} L^{\prime }\left(\tau \right)/2={\left(\tau -\tau_{0}\right)}^{2}+i\alpha \sigma^{2}\tau^{-2}=0 \end{array}$$

If we assume that the solution is τ = τ_0_ + δτ where δτ/τ_0_ ≪ 1,


$$\begin{array}{l} L^{\prime }\left(\tau_{0}+\delta \tau \right)/2=\delta \tau +i\alpha \sigma^{2}\tau_{0}^{-2}{\left(1+\frac{\delta \tau }{\tau_{0}}\right)}^{-2}\;. \end{array}$$

This leads to the approximation


$$\begin{array}{l} \;\left(1-2i\beta \right)\left(\frac{\delta \tau }{\tau_{0}}\right)\approx -i\beta \;, \end{array}$$

where β = ασ^2^$\tau_{0}^{-3}$ = 2πσ^2^$\tau_{0}^{-3}$ *t* is dimensionless. Since we can regard 0 ≤ *t* ≤ τ_0_, we have 0 ≤ β ≤ 2πσ^2^$\tau_{0}^{-2}$. Therefore, β ≪ 1 if σ^2^ ≪ $\tau_{0}^{2}$/2π. Then,


$$\begin{array}{l} \;\frac{\delta \tau }{\tau_{0}}\approx -\frac{i\beta }{1-2i\beta }\;. \end{array}$$

With a little algebra, we obtain


$$\begin{array}{l} L(\tau_{0}+\delta \tau )/\tau_{0}^{2}\;\;\approx \;\;{\left(\delta \tau /\tau_{0}\right)}^{2}-2i\beta {\left(1+\delta \tau /\tau_{0}\right)}^{-1}\\ \;\;\;\;\;\;\;\;\;\;\;\;\;\;\;\;\;\;\;\;\;\;\;\;\;\;\approx \;-2i\beta +\beta^{2}+2i\beta^{3}-\cdots \;. \end{array}$$

Therefore,


$$\begin{array}{l} \;I=\int d\tau e^{-L\left(\tau \right)/2\sigma^{2}}=\int due^{-L\left(\tau_{0}+\delta \tau +u\right)/2\sigma^{2}} \end{array}$$

which approximates to


$$\begin{array}{l} \;I\approx \int due^{-u^{2}/2\sigma^{2}-\left(-2i\beta +\beta^{2}+2i\beta^{3}\right)\tau_{0}^{2}/2\sigma^{2}} \end{array}$$

and gives the expression in equation (7).

### Numerical simulations

All numerical simulations were performed using Mathematica 13 (Wolfram Research, Champaign, IL). 30 sets of periods of 300 oscillators were generated from a Gaussian distribution at mean 24 h and various standard deviations (mostly 4 h for Figures 3, 4) at different random seeds. Simulation was performed for the 30 circadian cycles (corresponding to 30 days). Estimation of period from these simulated oscillators were performed using fast Fourier transform after discretization into 15-min sampling interval as introduced earlier (Myung et al., 2012). The order parameter was estimated at the end of the simulation duration.

## Data availability statement

The original contributions presented in the study are included in the article/supplementary material, further inquiries can be directed to the corresponding authors.

## Author contributions

JM conceptualized the study and wrote the first draft of the manuscript. JM, SH, and CS performed mathematical analysis. JM, HV, and M-YW interpreted the results. All authors discussed the results, contributed to the article and final manuscript, and approved the submitted version.
